# Mach-Zehnder Interferometric Immunosensor for Detection of Aflatoxin M1 in Milk, Chocolate Milk, and Yogurt

**DOI:** 10.3390/bios13060592

**Published:** 2023-05-30

**Authors:** Michailia Angelopoulou, Dimitra Kourti, Konstantinos Misiakos, Anastasios Economou, Panagiota Petrou, Sotirios Kakabakos

**Affiliations:** 1 Immunoassays/Immunosensors Lab, Institute of Nuclear & Radiological Sciences & Technology, Energy & Safety, NCSR “Demokritos”, 15341 Aghia Paraskevi, Greece; d.kourti@rrp.demokritos.gr (D.K.); ypetrou@rrp.demokritos.gr (P.P.); 2 Analytical Chemistry Lab, Department of Chemistry, University of Athens, Panepistimiopolis Zografou, 15771 Athens, Greece; aeconomo@chem.uoa.gr; 3 Institute of Nanoscience & Nanotechnology, NCSR “Demokritos”, 15341 Aghia Paraskevi, Greece; k.misiakos@inn.demokritos.gr

**Keywords:** integrated Mach–Zehnder interferometers, aflatoxin M1, milk, yogurt, optical sensor

## Abstract

Aflatoxin M1 (AFM1) is detected in the milk of animals after ingestion of aflatoxin B1-contaminated food; since 2002, it has been categorized as a group I carcinogen. In this work, a silicon-based optoelectronic immunosensor for the detection of AFM1 in milk, chocolate milk, and yogurt has been developed. The immunosensor consists of ten Mach–Zehnder silicon nitride waveguide interferometers (MZIs) integrated on the same chip with the respective light sources, and an external spectrophotometer for transmission spectra collection. The sensing arm windows of MZIs are bio-functionalized after chip activation with aminosilane by spotting an AFM1 conjugate with bovine serum albumin. For AFM1 detection, a three-step competitive immunoassay is employed, including the primary reaction with a rabbit polyclonal anti-AFM1 antibody, followed by biotinylated donkey polyclonal anti-rabbit IgG antibody and streptavidin. The assay duration was 15 min with limits of detection of 0.005 ng/mL in both full-fat and chocolate milk, and 0.01 ng/mL in yogurt, which are lower than the maximum allowable concentration of 0.05 ng/mL set by the European Union. The assay is accurate (% recovery values 86.7–115) and repeatable (inter- and intra-assay variation coefficients <8%). The excellent analytical performance of the proposed immunosensor paves the way for accurate on-site AFM1 determination in milk.

## 1. Introduction

Aflatoxin B1 (AFB1) is the most toxic naturally encountered mycotoxin. It is produced by *Aspergillus flavus* or *Aspergillus parasiticus* and detected in animals that have consumed contaminated feedstuffs [1]. Milk derived from these animals contains the hydroxylated metabolite of AFB1 produced in the liver of animals, namely aflatoxin M1 (AFM1) [2]. It has been determined that approximately 0.3–6.2% of the consumed AFB1 is transferred as AFM1 in mammals’ milk [3]. Thus, the consumption of milk contaminated with AFM1, and consequently of dairy products prepared from this milk, threatens people’s health due to the genotoxic, mutagenic, teratogenic, and carcinogenic properties of AFM1 [4]. More specifically, the International Agency for Research on Cancer (IARC) has categorized AFM1 as carcinogenic (Group 1) to humans [5] since it affects the liver, causing cirrhosis and hepatocellular carcinoma. Furthermore, long-term exposure to AFM1 can cause additional serious health problems, such as immunosuppression and nutritional dysfunctions. It should be also noted that high levels of AFM1 can cause stunted growth and delayed development in infants [6,7]. 

The implications in public health from the consumption of dairy products contaminated by AFM1 combined with its high stability during thermal processing, including cooking, pasteurization, or sterilization [6], render its detection in both raw milk and dairy products indispensable. Aiming to protect public health from dairy products contaminated with AFM1, maximum allowable concentrations for AFM1 in milk have been established by the regulatory authorities worldwide. In the EU, a limit of 0.05 ng/mL and 0.025 ng/mL has been set for adult and infant milk consumption, respectively [8], while the respective limit in other dairy products, such as yogurt, is 0.05 ng/mL [9]. On the other hand, in the USA, the USFDA has set a limit of 0.5 ng/mL for AFM1 in milk and dairy products [10]. 

To be able to monitor the AFM1 in raw milk and dairy products, the efficacy and reliability of the methods for the detection of AFM1 in these foods are of paramount importance. Thus, various chromatographic, molecular, immunological, and biochemical methods have been developed over the years for the determination of AFM1 levels in dairy products [11]. Thin-layer chromatography (TLC) [12] was the first technique used due to its low-cost and simplicity. Shortly afterwards, it was replaced by more sophisticated and sensitive chromatographic techniques, such as high-performance liquid chromatography coupled to a mass spectrometry (HPLC-MS) [13] or fluorescence detector (HPLC-FLD) [14,15] which have been established as reference methods. In addition, immunological methods such as enzyme-linked immunosorbent assays (ELISA) [16,17] and fluorescence [18] and chemiluminescent immunoassays [19] were introduced; they are characterized by high sensitivity, simple sample preparation, and lower cost of instrumentation compared with the chromatographic techniques. However, these techniques require trained personnel and cannot be performed at the point-of-need. To surpass this problem, immunochromatographic strips were developed for on-site analysis of AFM1 [20], providing however, semi-quantitative results. Thus, the quest for portable analytical devices led to the development of biosensors that can provide high sensitivity and specificity, fast analysis, and quantitative results usually in real-time [21]. In the last years, several types of biosensors based on electrochemical [22,23,24,25,26] or optical transducers [27,28,29,30,31,32,33,34] have been employed for the detection of AFM1. Amongst these, optical biosensors, based either on label or on label-free signal transduction principles, have considerable advantages over the electrochemical ones, as they exhibit high sensitivity, increased miniaturization potential, and less interference from the sample matrix. 

In this work, an optical immunosensor based on an array of Mach–Zehnder interferometers (MZIs) integrated monolithically on a silicon chip along with their respective light sources is employed for the sensitive detection of AFM1 in milk, chocolate milk, and baby yogurt samples. The proposed immunosensor [35] has already been applied for the detection of analytes related to food quality and safety such as goat milk adulteration with cow milk [36], mozzarella and feta cheese adulteration with cow milk [37], multiplex determination of allergens in food industry rinsing water [38], as well as bacteria detection in drinking water and milk [39]. However, in all the above cases, the targeted concentrations of the analytes, concerning especially milk and cheese, was relatively high allowing for sample dilution, therefore surpassing strong matrix effects. Here, given the very low concentrations of the targeted analyte (<50 pg/mL) in milk and dairy products, sample dilution could seriously affect the detection sensitivity. Therefore, in this work, for the first time, the proposed immunosensor was challenged through the determination of AFM1 in non-diluted or pretreated samples. For the detection, a three-step competitive immunoassay was adopted which efficiently surpassed sample matrix effects. All assay parameters, including AFM1-bovine serum albumin (AFM1-BSA) concentration for immobilization onto the chip, anti-AFM1 antibody concentration, assay configuration, and assay duration, have been optimized with respect to the required analytical characteristics.

## 2. Materials and Methods

### 2.1. Materials

Aflatoxin M1 (AFM1), bovine serum albumin (BSA), AFM1 conjugate with BSA (AFM1-BSA), donkey polyclonal anti-rabbit IgG antibody (secondary antibody), methanol HPLC grade, 2′,2-azino-bis(3-ethylbenzothiazoline-6-sulphonic acid) (ABTS), and (3-aminopropyl)triethoxysilane (APTES) were from Sigma-Aldrich (Darmstadt, Germany). Streptavidin and 3-Sulfo-succinimidyl-6-[biotinamido]hexanoate (Sulfo-NHS-LC-biotin) were from Thermo Fisher Scientific (Waltham, MO, USA). The polyclonal anti-AFM1 antibody developed in rabbits was from AntiProt (Puchheim, Germany). Biotinylation of donkey anti-rabbit IgG antibody was performed according to a literature protocol [32]. 

### 2.2. Chip Fabrication and Instrumentation

**Figure 1:**
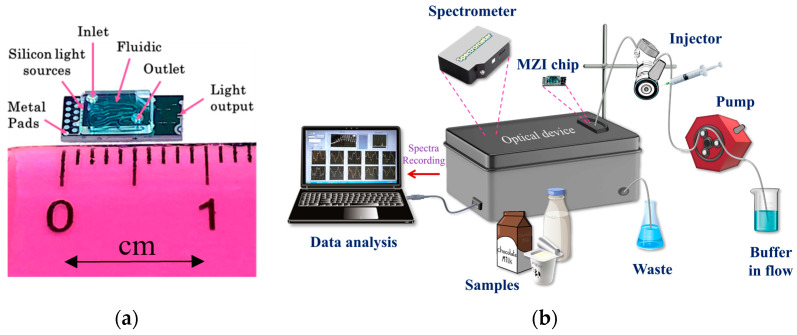
(**a**) Image of the MZI chip assembled with the microfluidic. (**b**) Schematic illustration of the measurement set-up.

The fabrication of the sensor chips consisting of 10 BB-MZIs integrated along with their respective light emitting diodes (LEDs) was performed as described previously [36,38] (Figure 1a). Details concerning the chip and the measurement set-up are described in the Supplementary Materials (Figure S1 and Description of the optofluidic chip and the measurement set-up). For the delivery of the reagents to the chip surface, a microfluidic cover with inlet and outlet holes was attached to the biofunctionalized chip. The chip was then placed in the docking station of the instrument to establish both fluidic and electrical connections. The delivery of the reagents was performed through a peristaltic pump and an injector (Rheodyne 7725i) with a 100 μL loop. The ten MZIs converged at the edge of the chip and the transmission spectra were directed via an optical fiber to a spectrometer (QE65000, Ocean Inside). During the assay, the spectrum of each waveguide was recorded every 10 s (1 s for each MZI of the chip), and the spectral shifts caused by the binding reactions were converted to phase shifts in TE polarization (analytical signal) through discrete Fourier transform. In Figure 1b, a 3D schematic of the instrumentation is provided.

### 2.3. Chip Activation

The chips were cleaned by immersion in acetone and isopropanol for 15 min under sonication, and then in Piranha solution (1:3 *v*/*v* H_2_SO_4_/30% *v*/*v* H_2_O_2_) for 30 s, followed by washing with doubly distilled water and drying with Ν_2_. The next step was the chemical activation of the chips through hydrophilization by O_2_ plasma for 30 s and immersion for 2 min in a 0.5% (*v*/*v*) APTES solution in distilled water. After washing, drying, and curing at 120 °C for 20 min, the chips were kept for 48 h in a desiccator. Then, the chips were spotted with the BioOdyssey Calligrapher Mini Arrayer as follows: 7 MZIs per chip (working sensors) were spotted with a 100 μg/mL of AFM1-BSA conjugate solution in 0.1 M carbonate buffer, pH 9.2, and the remaining 3 (reference sensors) with a 100 μg/mL BSA solution in the same buffer and incubated at 4 ^o^C overnight (Figure S1). Prior to use, the chips were washed with 50 mM Tris-HCl, pH 7.8, 0.9% (*w*/*v*) NaCl, to remove excess of spotted non-immobilized proteins, blocked for 2 h with 1% (*w*/*v*) BSA in 0.1 M NaHCO_3_ solution, pH 8.5, washed again, dried under nitrogen stream, and stored in a desiccator until use. 

### 2.4. Preparation of Calibrators/Samples

A 100 μg/mL AFM1 stock solution in highly pure methanol was used to prepare calibrators (0.01 to 2 ng/mL) in 0.05 M Tris-HCl buffer, pH 7.8, 0.5 % (*w*/*v*), 0.9% (*w*/*v*) NaCl (assay buffer). Pasteurized full-fat cow milk and baby yogurt (DELTA FOODS S.A.) as well as chocolate milk (EVOL S.A.) were obtained from local stores and were found not containing detectable amounts of AFM1 using an ELISA kit (AgraQuant^®^ Aflatoxin M1 High Sensitivity, Romer Labs GmbH; Butzbach, Germany). The detection limit of the kit was 2.9 pg/mL. Milk samples were fortified with AFM1 and analyzed without further treatment, whereas chocolate milk samples after spiking were centrifuged for 5 min at 3000× *g* and the supernatant was collected for analysis. Yogurt samples were treated after spiking following a published protocol [40]. In brief, 50 g of yogurt samples were spiked with appropriate amounts of AFM1 and then they were homogenized using a kitchen mixer. From each spiked yogurt sample, 2 g were mixed with 2 mL of a 7% *w*/*v* sodium citrate solution and stirred for 15 min at 50 °C. After cooling the mixture in an ice bath for 1 min, it was centrifuged for 15 min at 3000× *g*. The fatty upper layer was discarded, and the liquid serum was collected and analyzed. Taking into account that the water content of the yogurt was approximately 80%, a correction factor of 1.8 was applied to the AFM1 concentrations determined in the spiked samples.

### 2.5. AFM1 Detection with the MZI Immunosensor

**Figure 2:**
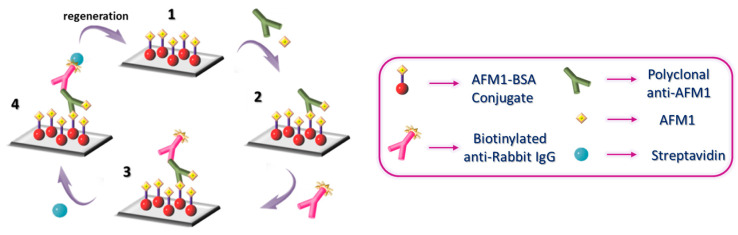
Schematic of the 3-step competitive assay for the determination of AFM1 showing: (1) AFM1-BSA conjugate immobilization, (2) immunoreaction with mixtures of AFM1 calibrators/samples and anti-AFM1 antibody, (3) reaction with the biotinylated donkey anti-rabbit IgG antibody, and (4) reaction with streptavidin followed by chip regeneration.

Prior to assay, the chip was equilibrated with 50 mM Tris-HCl, pH 7.8, 0.9% (*w*/*v*) NaCl (running buffer), and then 200 μL of 1:1 volume mixtures of calibrators/milk/yogurt samples with a 1 μg/mL rabbit polyclonal anti-AFM1 antibody solution in assay buffer were run over the chip at a rate of 35 μL/min (2 injections of 100 μL each). Then, running buffer was flown for 3 min followed by 100 μL of a 10 μg/mL biotinylated donkey anti-rabbit IgG antibody in assay buffer and 100 μL of a 10 μg/mL streptavidin solution in assay buffer. After equilibration with running buffer, the chip was regenerated by passing 100 μL of 0.5% *w*/*v* SDS-HCl solution, pH 1.3 (regeneration solution), followed again by equilibration with running buffer. A schematic of the analysis cycle is presented in Figure 2. The analytical signal is the difference of the phase shift value upon equilibration with running buffer after streptavidin to the value prior to the introduction of biotinylated secondary antibody. 

## 3. Results and Discussion

### 3.1. Optimization of Assay Parameters

**Figure 3:**
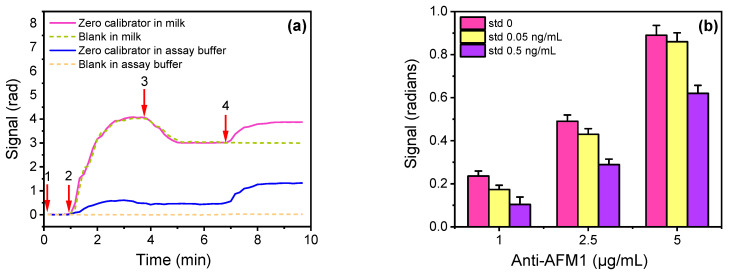
(**a**) Real-time signal responses obtained for an AFM1 zero calibrator in assay buffer (blue and dashed orange line) and in milk (purple and dashed green line) from MZIs spotted with AFM1-BSA (blue and purple line) or BSA (dashed orange and green line). The arrows indicate: washing buffer (1–2), mixture of zero calibrator in assay buffer/milk with a 5 μg/mL rabbit anti-AFM1 antibody (2–3), washing buffer (3–4), and a 10 μg/mL anti-rabbit IgG antibody (4-end). (**b**) Net signals obtained for zero calibrator (magenta columns) and calibrators containing 0.05 (yellow columns) or 0.5 ng/mL AFM1 (purple columns) versus the anti-AFM1 antibody concentration. The working MZIs were spotted with 200 μg/mL of AFM1-BSA and the reference ones with 200 μg/mL of BSA. Each point is the mean of 7 measurements ± SD.

AFM1 determination is based on a competitive immunoassay principle according to which an AFM1-BSA conjugate is immobilized onto the chip surface. As in all competitive immunoassays, the maximum signal is obtained for AFM1 zero calibrator and decreases as the amount of AFM1 in the sample increases. To achieve the lowest possible detection limit and meet EU legislation requirements, dilution of samples was avoided since it could negatively affect the assay sensitivity. However, from preliminary experiments where zero calibrator prepared in assay buffer or milk were assayed, it was found that it was not possible to monitor the reaction of rabbit anti-AFM1 antibody with the AFM1-BSA conjugate spotted onto the MZIs in the presence of milk (Figure 3a). In particular, the response obtained from the MZIs spotted with the AFM1-BSA conjugate (Figure 3a, purple line) could not be distinguished from the response of MZIs spotted with BSA (Figure 3a, dashed green line) even when the reaction mixture has been washed out from the sensor (Figure 3a, purple and dashed green line, arrow 3 to 4). This was attributed to the contribution on the sensor response of milk ingredients (lipids, proteins etc.) that bind non-specifically onto the MZI sensing window surface, since in the case of the zero calibrator in the buffer, there was a clear difference (approximately 0.5 rad) in the signal from MZIs spotted with AFM1-BSA conjugate with respect to that obtained from MZIs spotted with BSA (Figure 3a, blue and dashed orange line, arrow 3 to 4). For this reason, an assay configuration involving reaction with anti-rabbit IgG antibody (secondary antibody) after the primary immunoreaction was implemented. As shown in Figure 3a, very similar responses were obtained for both zero calibrator in the assay buffer and milk (arrow 4 to end) due to reaction with the anti-rabbit IgG antibody from the MZIs spotted with AFM1-BSA (working sensors), whereas there was no detectable response from the reference sensor. 

Based on this finding, optimization of the rabbit anti-AFM1 concentration in terms of the zero calibrator signal and assay sensitivity was performed for the 2-step assay format taking into account the signal obtained during the secondary immunoreaction. Hence, mixtures of zero calibrator in assay buffer with anti-AFM1 antibody solutions with concentration ranging from 1 to 5 μg/mL were passed over the chip for 3 min, followed by a washing step prior to reaction with 10 μg/mL secondary antibody for another 3 min. In Figure 3b, the net signals (mean working sensors values—mean reference sensors values) for zero calibrator and calibrators with concentrations of 0.05 and 0.5 ng/mL using anti-AFM1 antibody at concentrations between 1 and 5 μg/mL are depicted. As shown, the zero calibrator signal increased by increasing the anti-AFM1 concentration from 1 to 5 μg/mL; however, at the same time, the detection sensitivity was negatively affected. Although the highest sensitivity was achieved using the 1 μg/mL of antibody, the zero calibrator signal obtained for this concentration was inadequate for assay performance deteriorating its dynamic range.

**Figure 4:**
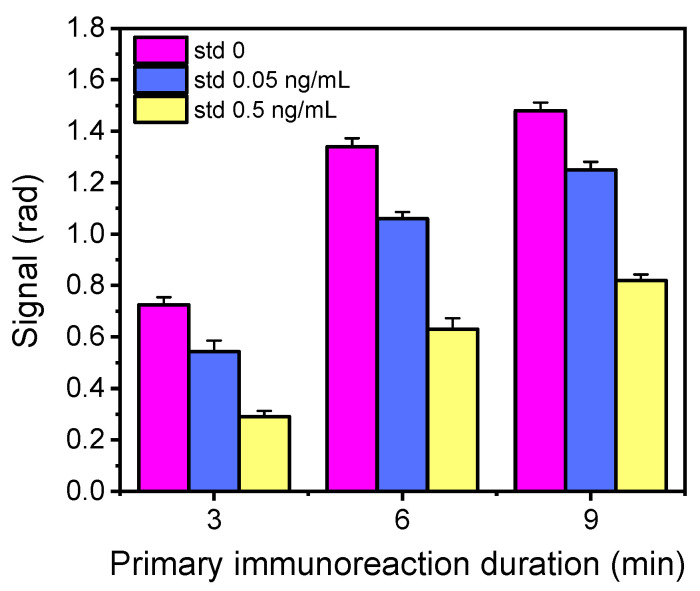
Net signals obtained for zero calibrator (magenta columns) and calibrators with concentrations of 0.05 (purple columns) or 0.5 ng/mL AFM1 (yellow columns) versus the primary immunoreaction duration. The working MZIs were spotted with 200 μg/mL of AFM1-BSA and the reference ones with 200 μg/mL of BSA; the primary antibody concentration was 1 μg/mL; the biotinylated secondary antibody and streptavidin concentration were 10 μg/mL. Each point is the mean of 7 measurements ± SD.

To surpass this problem, it was decided to increase the primary immunoreaction volume and to introduce a third assay step by employing a biotinylated secondary antibody instead of the non-biotinylated one, followed by reaction with streptavidin so as to enhance the signal. Thus, different sample volumes were run over the chip (100 to 300 μL through multiple injections of 100 μL each), increasing thus the time of the primary immunoreaction from 3 to 9 min. The reaction time with the biotinylated secondary antibody and the streptavidin was 3 min each. In Figure 4, the net chip signals obtained for the zero calibrator and calibrators with concentrations of 0.05 and 0.5 ng/mL following the 3-step assay versus the primary immunoreaction duration are provided. As shown, the introduction of streptavidin increased the signal 3 times compared with that received from the 2-step assay configuration (Figure 3b). Furthermore, when increasing the primary immunoreaction time from 3 to 6 min, the zero calibrator signals increased approximately 85% without compromising the assay sensitivity. The net zero calibrator signal obtained with the 3-step assay and primary immunoreaction time of 6 min is satisfactory for the assay performance. Further increase of the primary immunoreaction time to 9 min marginally increased the analytical signal and negatively affected the detection sensitivity. Hence, the three-step assay configuration with a 1 μg/mL anti-AFM1 antibody concentration and 15 min total assay duration (6 min for the primary reaction, 3 min washing, 3 min for the biotinylated secondary antibody, and 3 min streptavidin) was adopted.

Using the 3-step assay configuration, the optimum concentration of AFM1-BSA for immobilization to the MZI sensing window was determined. Hence, AFM1-BSA solutions with concentrations ranging from 10 to 500 μg/mL were spotted to different chips and tested by running mixtures of zero calibrator with a 1 μg/mL anti-AFM1 solution for 6 min, biotinylated secondary antibody for 3 min, and streptavidin for another 3 min. Similarly, a 0.05 ng/mL AFM1 calibrator was also tested. As is depicted in Figure S2, the zero calibrator signal reached maximum plateau values for AFM1-BSA concentrations equal to or higher than 100 μg/mL. Furthermore, the percent signal received for the 0.05 ng/mL AFM1 calibrator with respect to zero signal was approximately 70% for chips functionalized with concentrations of AFM1-BSA lower than or equal to 100 μg/mL and increased for higher concentrations. Consequently, the AFM1-BSA concentration of 100 μg/mL, that provided maximum plateau zero calibrator signal and optimal sensitivity, was selected for coating of the chips.

### 3.2. Matrix Effect of Milk, Chocolate Milk and Yogurt

Milk is a matrix characterized by high opacity; it also contains high amounts of various proteins and fats that could affect the analytical performance of an optical biosensor. This phenomenon is intensified for chocolate milk due to its cocoa powder content and the consequent coloring. Yogurt is also a very particular matrix that contains part of the milk proteins and fats and in addition has an acidic pH value due to the conversion of milk lactose to lactic acid during milk fermentation that leads to yogurt. 

**Figure 5:**
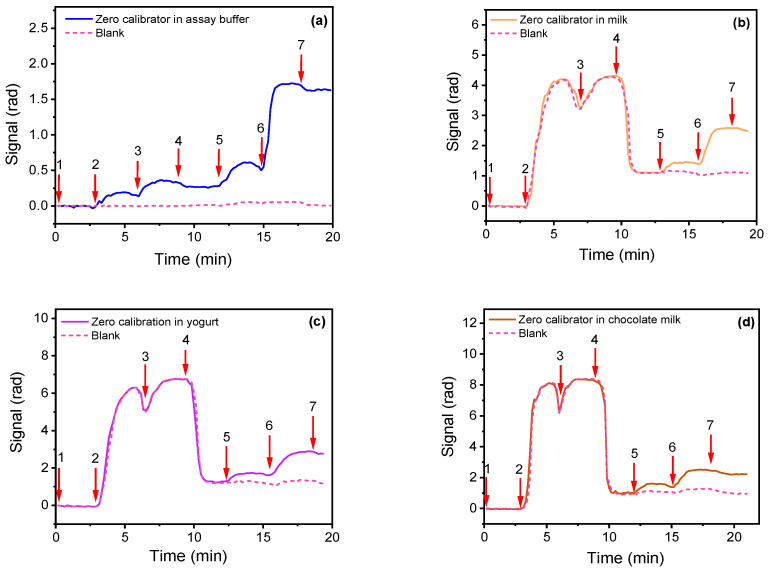
Real time responses for zero calibrators in (**a**) assay buffer, (**b**) full-fat cow milk, (**c**) baby yogurt, and (**d**) chocolate milk. The arrows indicate: washing solution (1–2), zero calibrator/anti-AFM1 antibody mixture (2–4), washing solution (4–5), biotinylated secondary antibody (5–6), streptavidin (6–7), and washing solution (7-end). Dashed lines indicate the signal response corresponding to non-specific binding (blank).

In Figure 5, the real-time signals for zero calibrator in assay buffer (Figure 5a), full-fat cow milk (Figure 5b), chocolate milk (Figure 5c), and baby yogurt (Figure 5d), respectively, are presented. An ELISA kit was employed to confirm absence of detectable AFM1 amounts in the three matrices used. 

As shown in Figure 5b, the presence of milk resulted in an abrupt increase in sensor response (arrow 2 to 4) that could be attributed to its opacity that affected the transmitted light but also to non-specific binding of proteins and fats present in milk onto the sensor surface. The latter is indicated by the fact that after washing out of the milk, the signal difference is approximately 5 times higher than the signal difference due to primary immunoreaction in assay buffer (Figure 5a, arrow 2 to 5). Nonetheless, the response obtained upon reaction with biotinylated secondary antibody and streptavidin (arrow 5 to 7) was identical to the respective signal obtained for the zero calibrator in buffer (Figure 5a, arrow 5 to 7). Regarding yogurt, the analysis of solution obtained following the extraction procedure described in the Materials and Methods section provided similar results for the zero calibrator with those obtained in buffer (Figure 5c).

On the other hand, concerning chocolate milk, even after centrifugation of the samples, a marginal effect onto the zero calibrator signal (approximately 10% lower than that received for assay buffer) was observed (Figure 5d). Therefore, for the determination of AFM1 in milk and yogurt, the calibrators could be prepared in assay buffer, whereas for the detection of AFM1 in chocolate milk, matrix-matched calibrators should be used to avoid bias when analyzing unknown samples. In all cases, for the preparation of the calibration curves, the analytical signal used was the difference of the phase shift value upon equilibration with running buffer after streptavidin to its value prior to the introduction of biotinylated secondary antibody minus the relevant non-specific binding values (provided by the waveguides coated with BSA). 

### 3.3. Analytical Characteristics

**Figure 6:**
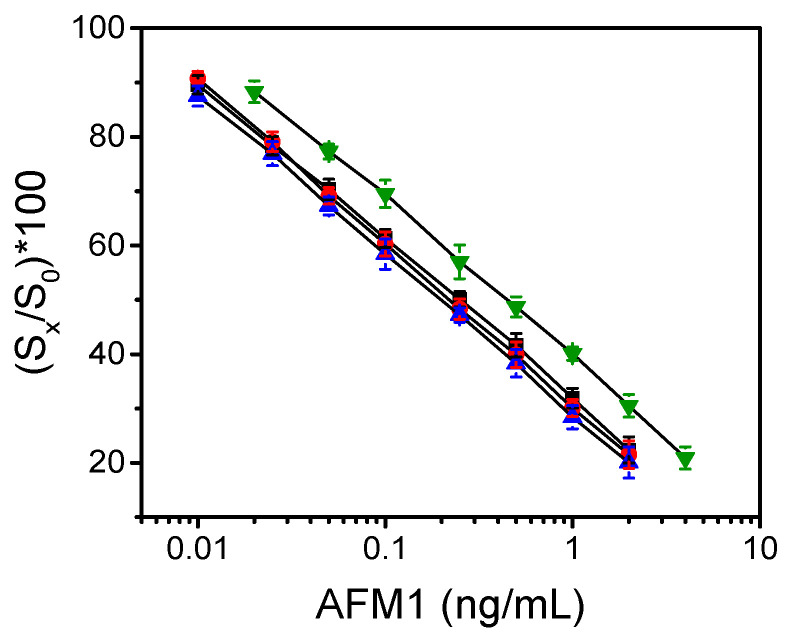
Typical AFM1 calibration curves in assay buffer (black squares), milk (red circles), chocolate milk (blue up triangles), and yogurt (green down triangles), obtained with the MZI immunosensor. Each point is the mean of 7 measurements ± SD.

In Figure 6, calibration curves received using calibrators prepared in the different matrices are depicted. In all cases, the calibration curves were plotted as percent ratios of signals corresponding to AFM1 calibrators (Sx) with respect to the zero calibrator signal (S_0_). Real-time responses obtained for the different calibrators in milk are also provided in Figure S3.

As is shown, the calibration curves obtained with calibrators prepared in milk and chocolate milk were almost superimposed with that obtained using calibrators in assay buffer. On the other hand, the calibration curve obtained with calibrators prepared in yogurt was parallel displaced to higher dynamic range, in accordance with the dilution that occurred due to the sample preparation procedure.

The detection limit (LOD) for AFM1 in all matrices tested was calculated as the concentration corresponding to signal equal to -3SD of the mean zero calibrator signals (21 replicate values from 3 chips; 7 MZIs per chip). Considering the coefficients of variation determined for the zero calibrator (1.8–2.0%) for all sample matrices, the calculated LODs for milk and chocolate milk was 0.005 ng/mL, whereas that for yogurt was 0.01 ng/mL (due to dilution for the extraction). These LODs are below the respective maximum allowable limits set by EU for AFM1 in milk for adults (0.05 ng/mL), milk for infants (0.025 ng/mL), and yogurt (0.05 ng/mL), respectively. The working range of AFM1 assay in milk matrices ranged from 0.01 to 2.0 ng/mL and in yogurt from 0.02 to 4.0 ng/mL. 

The intra-assay variation coefficients (CVs) were determined by analyzing milk, chocolate milk, and yogurt samples fortified with three different concentrations of AFM1 (0.04, 0.4, 1.2 ng/mL) three times in the same day. Furthermore, the inter-assay CVs, determined through measurements of the same samples in 10 random days for 2 months, were less than 4.5% and 8%, respectively, indicating the excellent repeatability of the assay.

The accuracy of the assay was assessed through recovery experiments, in which samples of pasteurized cow milk and chocolate milk were spiked with AFM1 at concentrations of 0.02, 0.2, and 1.5 ng/mL, whereas for yogurt, samples were fortified at 0.04, 0.4, and 3 ng/mL final AFM1 concentrations. Each sample was analyzed in triplicate and the %recovery was calculated according to the equation:

$$\%Recovery=\frac{AFM1\;concentration\;determined}{\begin{array}{c} AFM1\;concentration\;added \end{array}}\times 100$$

As shown in Table S1, the recovery values ranged from 86.7 to 115%, confirming the method accuracy.

The specificity of the assay was determined through cross-reactivity experiments against other aflatoxins with similar chemical structure with AFM1 such as aflatoxin B_1_ (AFB_1_), aflatoxin B_2_ (AFB_2_), and aflatoxin G_1_ (AFG_1_). As shown in Figure S4, the cross-reactivities determined were 1.2, 0.21, and 0.09% for AFB_1,_ AFB_2_, and AFG_1,_ respectively, indicating the high specificity of the proposed immunosensor regarding other aflatoxins with similar molecular structure compared with AFM1.

### 3.4. Sensor Regeneration and Reuse

The possibility of sensor surface regeneration, i.e., the removal of antibodies bound to the antigen onto the chip surface by passing a regeneration solution over the functionalized chip, and its reuse was investigated. For this purpose, after the completion of an assay cycle, different solutions including an immunochromatography column elution buffer, 0.5% (*w*/*v*) SDS-HCl solution, pH 1.3, 50 mM HCl, 50 mM NaOH, or 50 mM HCl followed by 50 mM NaOH were passed over the chip for 3 min each (Figure S5a). To determine the percentage of anti-AFM1 antibody remaining on the chip surface after regeneration, biotinylated anti-rabbit antibody and streptavidin were flown over the chip and the signal obtained was compared to that of the zero calibrator. It was found that the use of SDS-HCl solution removed almost completely the bound anti-AFM1 antibody (less than 1.5% of the respective zero calibrator signal). The other solutions tested did not remove the antibody bound to the immobilized AFM1-conjugate as efficiently. In addition, employing the SDS-HCl solution for chip regeneration, the zero calibrator response prior to (Figure S5a, purple columns) and after the regeneration (Figure S5a, magenta columns) was identical, indicating that regeneration did not affected the immobilized AFM1-BSA. Real-time signal response obtained for two consecutive assay/regeneration cycles is provided in Figure S6. The other solutions tested, especially the alkaline ones, affected the immobilized AFM1 conjugate to different degrees, leading to signal loss after regeneration. Using the SDS-HCl solution for regeneration, the stability of the functionalized MZI immunosensor chips against repetitive immunoassay/regeneration cycles was also evaluated. As shown in Figure S7, chips spotted with AFM1-BSA could be regenerated up to 15 times using the SDS-HCl solution, without any signal decrease since all values lay within the mean value ± 2SD signal range.

### 3.5. Comparison with Literature Optical Immunosensors for AFM1 Detection in Milk and Dairy Products

A comparison of optical immunosensors either employing labels or label-free ones reported in the literature for the determination of AFM1 in milk and dairy products with the MZI immunosensor developed, with respect to the detection principle and the label used, the sample matrix along with sample preparation employed, the LODs, and the analysis time required, is provided in Table 1. 

Regarding the detection of AFM1 with optical immunosensors that employ labels, a long-range surface plasmon-enhanced fluorescence spectroscopy sensor provided a 10-time lower LOD (0.6 pg/mL) compared with that of the proposed immunosensor; however, the assay duration was 3.5 times longer [41]. Compared with an evanescent-wave fiber-optic sensor employing a fluorescent label and applied to detect AFM1 in matrices such as milk, cheese, and milk tea [42], the proposed MZI sensor was 10 times more sensitive. Although the duration of the assay with the reported sensor was 2-fold shorter (8 min) compared with the developed one, a 35-min pretreatment of the sample, including extraction with organic solvents and 20-time dilution of the extract was required, increasing the total analysis time to 43 min. Karczmarczyk et al. developed an SPR immunosensor, implementing gold nanoparticles as labels [28]. This sensor provided a 3.5 time higher LOD in a 3.6 time longer assay duration compared with the proposed immunosensor. A planar waveguide fluorescence immunosensor (MPWFI), which used extracted and 50-time diluted milk for the analysis, has been reported in the literature [43]. Compared with that, the proposed immunosensor presented an LOD that was 11 times lower in a similar assay time (17 min).

Concerning the reported label-free immunosensors for AFM1 detection, the proposed sensor achieved comparable detection sensitivity with an immunosensor based on white light reflectance spectroscopy (WLRS), which had a longer analysis time of 25 min instead of the 15 min of the proposed one [32]. In 2019, an immunosensor based on Fab functionalized Si_3_N_4_ asymmetric MZI for AFM1 detection in milk was reported [29]. The assay was extremely fast (1.5 min); however, this was achieved following a quite long multi-step sample preparation procedure, including defatting, 20-time pre-concentration of the sample, and purification and detection of AFM1 in the purified eluates. Even in that case, the LOD achieved was 3 times higher (16.8 pg/mL) compared with that provided by the proposed sensor. Finally, a label-free SPR sensor for AFM1 determination in milk and milk powder was also reported [31]. The sensor exhibited a 20 time higher LOD than that of the MZI immunosensor developed with an assay duration of 10 min, although defatting and immunoaffinity separation of AFM1 was required prior to analysis. Overall, the proposed immunosensor is one of the fastest label-free optical immunosensors reported in the literature and amongst the most sensitive for the determination of AFM1 in milk avoiding any sample pretreatment. Furthermore, the MZI sensor is capable of detecting AFM1 in chocolate milk and yogurt with minimal sample preparation procedure.

## 4. Conclusions

A fast and ultrasensitive method for AFM1 determination in milk, chocolate milk, and yogurt employing arrays of MZIs monolithically integrated onto silicon chips along with their light sources was presented for the first time. The detection limit achieved was 10 times lower than the maximum allowable concentration set by the EU for milk for adults’ consumption and chocolate milk and 5 times lower for yogurt and milk for infants. Regarding the detection of AFM1 in milk, no sample pretreatment was required, whereas for chocolate milk a 5-min centrifugation was implemented. For analysis of yogurt samples, a quite simple extraction protocol reported in the literature that does not involve the use of organic solvents was found to be very efficient with respect to analyte recovery. Following the 3-step assay configuration, the strong matrix effect observed for both milk and yogurt samples was eliminated. The chip could be successfully regenerated and reused for up to 15 times, without any signal loss, thus reducing the total analysis cost. Moreover, the chip, if appropriately functionalized with immobilization of different recognition molecules on the ten different integrated MZIs, can be applied in addition to AFM1 for the simultaneous determination of more than one milk contaminant such as antibiotics, melamine, etc., in various dairy products. The small size of the chip, its multiplexing capabilities, the fast analysis time, and the excellent analytical characteristics achieved allow for the development of small-size portable instruments for the detection of analytes of interest at the point-of-need. 

## Figures and Tables

**Table 1:** Comparison of the MZI immunosensor developed with other optical immunosensors for determination of AFM1 in milk and dairy products.

Detection Principle	Label	Sample	LOD (pg/mL)	Assay Time (min)	Ref.
Long-range surface plasmon-enhanced fluorescence spectroscopy	Cy5-GaR	Milk (defatted)	0.6	53	[41]
Evanescent-wave fiber-optic	Cy5.5	Milk, Cheese, Milk tea (extraction with organic solvents and 20 times extract dilution)	50	8	[42]
Surface Plasmon Resonance (SPR)	Au NPs	Milk (defatted)	18	55	[28]
Planar waveguide fluorescence (MPWFI)	Cy5.5	Milk (extracted and 50 times diluted milk)	55	17	[43]
White Light Reflectance Spectroscopy (WLRS)	-	Milk	6	25	[32]
Fab functionalized Si_3_N_4_ Asymmetric Mach-Zehnder Interferometer (MZI)	-	Milk (defatted, 20 times pre-concentrated/purified eluents)	16.8	1.5	[29]
Surface Plasmon Resonance (SPR)	-	Milk/ Milk powder (defatted, immunoaffinity separation of AFM1 prior to analysis)	100	10	[31]
Mach–Zehnder Interferometer (MZI)	-	Milk, chocolate milk Yogurt	5 10	15	This work

## Data Availability

The data presented in this study are available on request from the corresponding author. The data are not publicly available due to privacy issues.

## Supplementary Materials

The following supporting information can be downloaded at: https://www.mdpi.com/article/10.3390/bios13060592/s1, Figure S1: Schematic of the chip indicating the arrangement of the LEDs, the array of the 10 MZIs, the fluidic outline and the spotting layout; Description of the optofluidic chip and the measurement set-up; Figure S2: Optimization of AFM1-BSA concentration for immobilization to working MZIs sensing windows; Figure S3: Real-time signal responses obtained for AFM1 calibrators prepared in milk employing the 3-step assay configuration; Experimental of the cross-reactivity study; Figure S4: Cross reactivity study results; Figure S5: Selection of the regeneration solution; Figure S6: Real-time signal response obtained for the AFM1 zero calibrator in assay buffer following two consecutive assays including regeneration with SDS-HCl solution; Figure S7: Stability of the sensor against repetitive regeneration/assay cycles.
