# Supplementary material for: Mach-Zehnder Interferometric Immunosensor for Detection of Aflatoxin M1 in Milk, Chocolate Milk, and Yogurt
Source: Biosensors (Basel). 2023 May 30;13(6):592. doi: 10.3390/bios13060592 (PMC10296510; doi:10.3390/bios13060592)
Supplement: Supplementary file 1 [file biosensors-13-00592-s001.zip › biosensors-2406159-supplementary.pdf]

biosensors
MDPI

Supplementary Materials

Mach-Zehnder interferometric immunosensor for detection of Aflatoxin M1 in milk, chocolate milk, and yogurt

Michailia Angelopoulou[1,*], Dimitra Kourti[1,2], Konstantinos Misiakos[3], Anastasios Economou[2], Panagiota Petrou[1], Sotirios Kakabakos[1,*]

[1] Immunoassays/Immunosensors Lab, Institute of Nuclear & Radiological Sciences & Technology, Energy & Safety, NCSR "Demokritos" Aghia Paraskevi 15341, Greece

[2] Analytical Chemistry Lab, Department of Chemistry, University of Athens, Panepistimiopolis Zografou 15771, Greece

[3] Institute of Nanoscience & Nanotechnology, NCSR "Demokritos", Aghia Paraskevi 15341, Greece

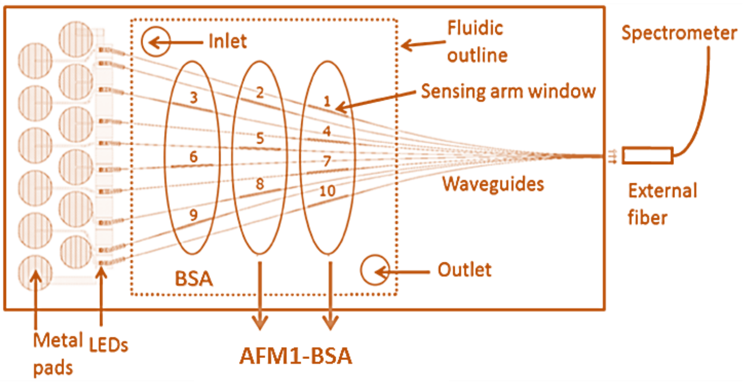

Figure S1. Schematic of the chip indicating the arrangement of the LEDs, the array of the 10 MZIs, the fluidic outline and the spotting layout.

Description of the optofluidic chip and the measurement set-up

A custom-designed docking station which provided both the electrical and fluidic connections was included in the measurement set-up [Anal. Chem. 2018, 90, 9559–9567]. An external optical fiber aligned to the docking station collected the transmission spectra from the edge of the chip and transferred them to a QE65000miniaturized spectrometer (Ocean Optics; Dunedin, FL). The spectrometer was synchronized by an electrical multiplexer which turned on/off the ten LEDs of the BB-MZI sequentially every 1 s. Thus, the spectrum from each BB-MZI was recorded every 10 s by the PC, which controlled the measuring apparatus. The solutions were run over the biofunctionalized chip through a microfluidic module made of a 800 μm thick poly(methyl methacrylate) (PMMA) foil with fluid inlet and outlet holes. The fluidic cell was created through photolithography of a laminated on the PMMA 75 μm thick dry film photoresist (Pyralux PC1015, DuPont) that also provided the seal to the chips acting as a pressure sensitive adhesive layer [Anal. Chem. 2018, 90, 9559–9567]. The chips were assembled with the fluidic module and inserted to the docking station of

the measuring apparatus; reagents were delivered using a peristaltic pump and an injector (Rheodyne 7725i) equipped with a 100 µL loop placed between the pump and the docking station.

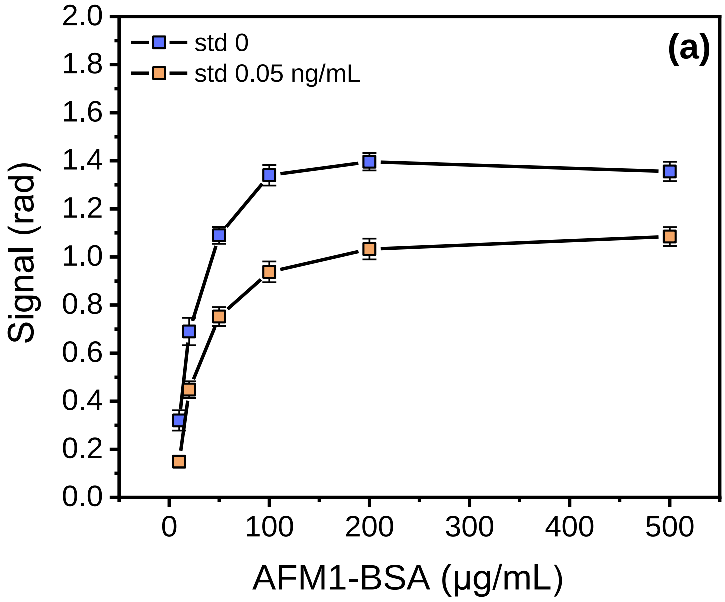

Figure S2. Optimization of AFM1-BSA concentration for immobilization to working MZIs sensing windows. The MZIs responses for the zero calibrator (blue points) and a calibrator containing 0.05 ng/mL AFM1 (orange points) are shown. Each point is the mean of 7 measurements ± SD.

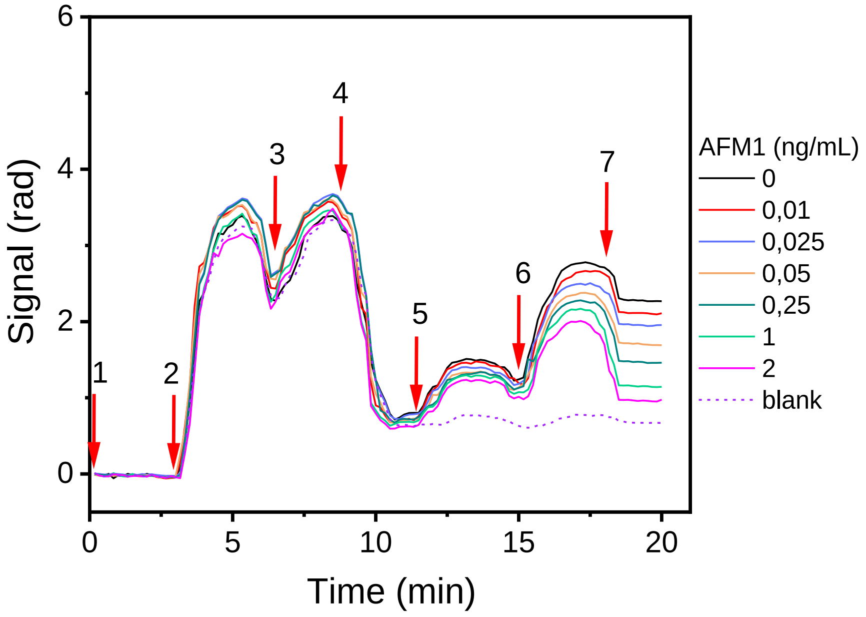

Figure S3. Real-time signal responses obtained for AFM1 calibrators in milk with concentrations ranging from 0 to 2 ng/mL employing the 3-step assay configuration. Dashed line indicates the signal response corresponding to non-specific binding (blank).

Table S1. Recovery of known amounts of AFM1 spiked in full-fat cow milk, yogurt, and chocolate milk samples.

Sample	Amount added (ng/mL)	Amount determined (ng/mL)	%Recovery
Cow milk	0.02	0.022	110%
	0.2	0.18	90.0%
	1.5	1.35	90.0%
Yogurt	0.04	0.036	90.0%
	0.4	0.46	115%
	3.0	3.39	113%
Chocolate milk	0.02	0.021	106%
	0.2	0.18	90.0%
	1.5	1.30	86.7%

Experimental of the cross-reactivity study

A crucial parameter that can jeopardize the assay performance is the specificity of the MZI immunosensor. For this reason, cross-reactivity experiments involving other aflatoxins with similar chemical structure with AFM1 such as aflatoxin B1 (AFB1), aflatoxin B2 (AFB2), and aflatoxin G1 (AFG1) were performed. Thus, stock solutions of the above aflatoxins were used for the preparation of solutions with concentration at the range of 0.1 to 1000 ng/mL in assay buffer, and run over the chip as described in Section 2.5. The percent cross-reactivity (%CR) was calculated from the calibration curves obtained with the tested cross-reactants with respect to the calibration curve o AFM1 according to the equation:

$$\%CR = \frac{IC50AFM1}{IC50\ cross\ reactant} * 100$$

where IC50 AFM1 is the concentration of AFM1 providing 50% inhibition and IC50 cross-reactant is the concentration of the tested aflatoxin corresponding to 50% inhibition of the respective zero calibrator signal. As shown in Figure S4, the cross-reactivities determined were 1.2% for AFB1 and 0.21% and 0.09% for AFB2 and AFG1, respectively, indicating the high specificity of the proposed immunosensor regarding other aflatoxins with similar molecular structure compared with AFM1.

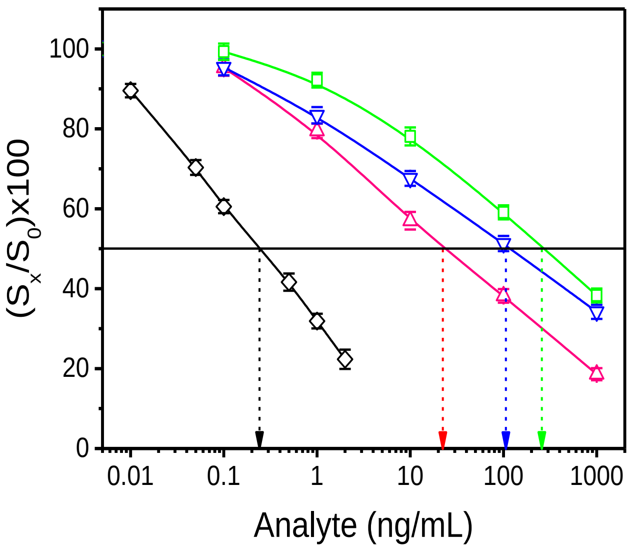

Figure S4. Cross-reactivity study results. Calibration curves of AFM1 (black cycles), AFB1 (pink up triangles), AFB2 (blue down triangles), and AFG1 (green squares) obtained from MZI immunosensor coated with AFM1-BSA conjugate. The dashed vertical lines correspond to analyte concentration that provides 50% inhibition (horizontal dashed black line).

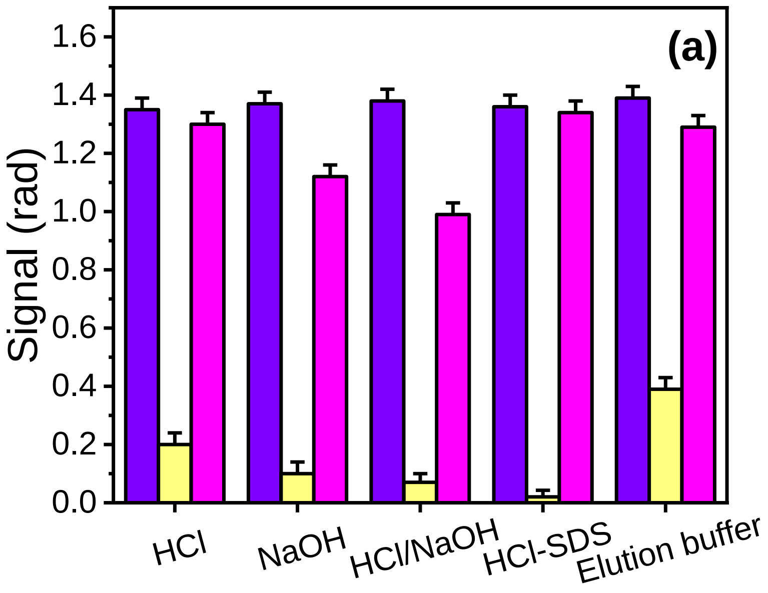

Figure S5. Selection of the regeneration solution. Sensor responses obtained for zero calibrator prior to regeneration (purple columns), the respective signals obtained upon running the biotinylated anti-rabbit IgG antibody and the streptavidin after treatment with different regeneration solutions (yellow columns), and zero calibrator signals after regeneration (magenta columns). Each point is the mean of 7 measurements ± SD.

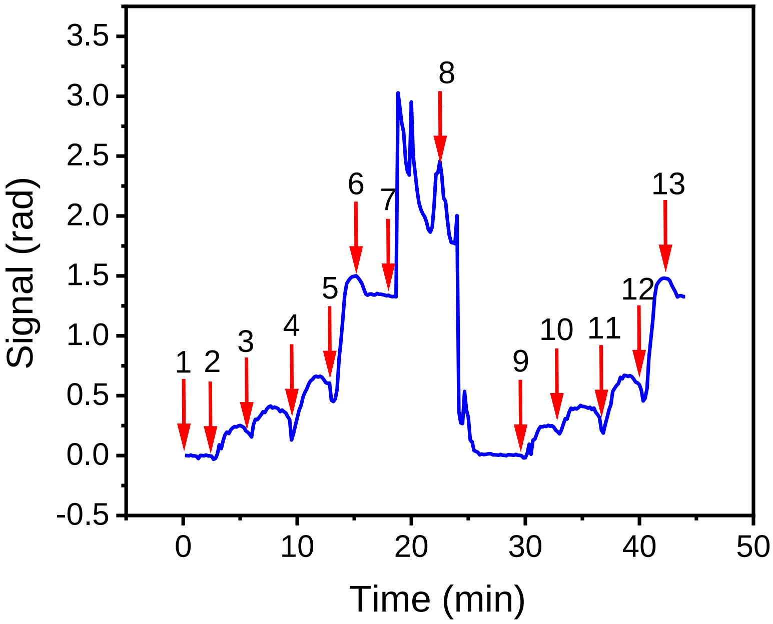

Figure S6. Real-time signal response obtained for the AFM1 zero calibrator in assay buffer following two consecutive assays including regeneration with SDS-HCl solution. The arrows show the sequence of solutions running over the chip as follows: washing solution (1-2), zero calibrator in assay buffer (2-4), biotinylated secondary antibody (4-5), streptavidin (5-6), washing solution (6-7), SDS-HCl (7-8), washing solution (8-9), zero calibrator prepared in assay buffer (9-11), biotinylated secondary antibody (11-12), streptavidin (12-13), and washing solution (13-end).

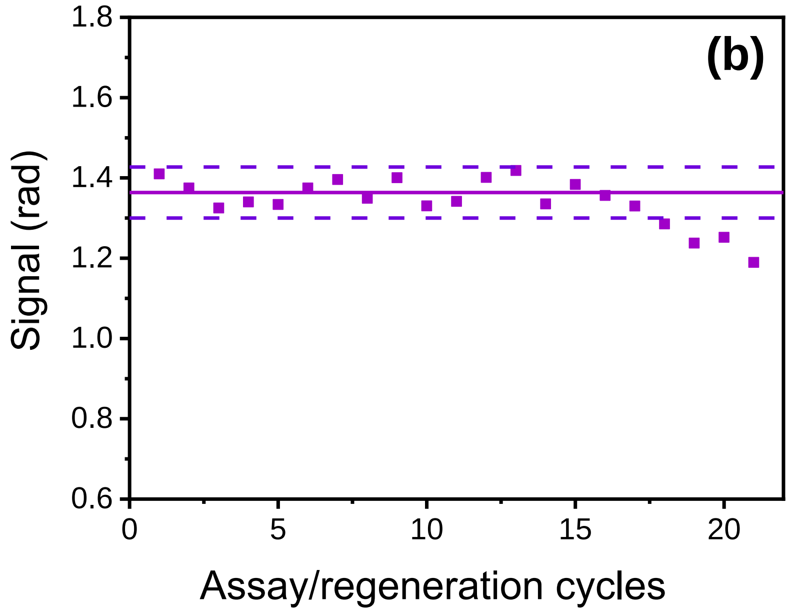

Figure S7. Stability of the sensor against repetitive regeneration/assay cycles. Sensor responses obtained for zero calibrator after repetitive regeneration/assay cycles with SDS-HCl, pH 1.3. Each point is the mean of 7 measurements ± SD.
